# Multiomic analysis of monocyte-derived alveolar macrophages in idiopathic pulmonary fibrosis

**DOI:** 10.1186/s12967-024-05398-y

**Published:** 2024-06-27

**Authors:** Miaomiao Zhang, Jinghao Zhang, Haisheng Hu, Yuan Zhou, ZhiWei Lin, Hui Jing, Baoqing Sun

**Affiliations:** 1 Department of Clinical Laboratory, National Center for Respiratory Medicine, National Clinical Research Center for Respiratory Disease, State Key Laboratory of Respiratory Disease, Guangzhou Institute of Respiratory Health, The First Affiliated Hospital of Guangzhou Medical University, Guangzhou, China; 2 Department of Internal Medicine II, University Hospital Bonn, Section of Pneumology, Bonn, Germany; 3 Department of Respiratory and Critical Care Medicine, Xuzhou Central Hospital, Xuzhou, China; 4 Department of Medicine II, Heart Center Bonn, University Hospital Bonn, Bonn, Germany; 5 Guangzhou Laboratory, Guangzhou, 510005 China

**Keywords:** Idiopathic pulmonary fibrosis, Multiomic, Monocyte-derived alveolar macrophages, Transcription factors

## Abstract

**Background:**

Monocyte-derived alveolar macrophages (Mo_AMs) are increasingly recognised as potential pathogenic factors for idiopathic pulmonary fibrosis (IPF). While scRNAseq analysis has proven valuable in the transcriptome profiling of Mo_AMs, the integration analysis of multi-omics may provide additional dimensions of understanding of these cellular populations.

**Methods:**

We performed multi-omics analysis on 116 scRNAseq, 119 bulkseq and five scATACseq lung tissue samples from IPF. We built a large-scale IPF scRNAseq atlas and conducted the Monocle 2/3 as well as the Cellchat to explore the developmental path and intercellular communication on Mo_AMs. We also reported the difference in metabolisms, tissue repair and phagocytosis between Mo_AMs and tissue-resident alveolar macrophages (TRMs). To determine whether Mo_AMs affected pulmonary function, we projected clinical phenotypes (FVC%pred) from the bulkseq dataset onto the scRNAseq atlas. Finally, we used scATATCseq to uncover the upstream regulatory mechanisms and determine key drivers in Mo_AMs.

**Results:**

We identified three Mo_AMs clusters and the trajectory analysis further validated the origin of these clusters. Moreover, via the Cellchat analysis, the CXCL12/CXCR4 axis was found to be involved in the molecular basis of reciprocal interactions between Mo_AMs and fibroblasts through the activation of the ERK pathway in Mo_AMs. SPP1_RecMacs (RecMacs, recruited macrophages) were higher in the low-FVC group than in the high-FVC group. Specifically, compared with TRMs, the functions of lipid and energetic metabolism as well as tissue repair were higher in Mo_AMs than TRMs. But, TRMs may have higher level of phagocytosis than TRMs. *SPIB (PU.1)*, *JUNB*, *JUND*, *BACH2*, *FOSL2*, and *SMARCC1* showed stronger association with open chromatin of Mo_AMs than TRMs. Significant upregulated expression and deep chromatin accessibility of APOE were observed in both SPP1_RecMacs and TRMs.

**Conclusion:**

Through trajectory analysis, it was confirmed that SPP1_RecMacs derived from Monocytes. Besides, Mo_AMs may influence FVC% pred and aggravate pulmonary fibrosis through the communication with fibroblasts. Furthermore, distinctive transcriptional regulators between Mo_AMs and TRMs implied that they may depend on different upstream regulatory mechanisms. Overall, this work provides a global overview of how Mo_AMs govern IPF and also helps determine better approaches and intervention therapies.

**Supplementary Information:**

The online version contains supplementary material available at 10.1186/s12967-024-05398-y.

As the most common interstitial lung disease, idiopathic pulmonary fibrosis (IPF) is a rapidly progressive and lethal respiratory disease without a precise aetiology. It affects 3–9 out of every 100,000 people worldwide, and the prevalence is rising globally [1, 2]. The few pharmacotherapies available have shown limited efficacy, and the overall 5-year survival rate of IPF remains low [3, 4, 65]. Lung transplantation is currently the only short-term therapy. Typical IPF characteristics include the formation of fibrosis niches, deposition of collagen, and expansion of the extracellular matrix, leading to restrictive ventilation impairment [5]. Thus, researchers have placed more emphasis on taking advantage of state-of-the-art technologies that could help uncover the central molecular mechanism of IPF.

As the most numerous immune cells in the lung, macrophages are divided into two types based on their location: alveolar macrophages (AMs), which act as the first-line defenders; and interstitial macrophages, which reside near the vasculature and interstitium. AMs are further divided by origin into monocyte-derived alveolar macrophages (Mo_AMs) and tissue-resident alveolar macrophages (TRMs). Recently, awareness has been mounting that Mo_AMs are the main factors in fibrotic development [6, 7]. Here, we ask why Mo_AMs, not TRMs are the main risk factors for fibrosis. What is the difference of upstream regulatory mechanisms between Mo_AMs and TRMs? scATACseq is a developing advanced technique to study epigenetic regulation and chromatin accessibility. Additionally, scATACseq has enabled a more thorough understanding of gene regulatory mechanisms that scRNAseq fails to capture. Thus, this technology could help us seek the answers of the above questions.

Previous studies have also demonstrated a positive feedback loop between Mo_AMs and fibroblasts. Mo_AMs have been found to promote the conversion from fibroblasts to myofibroblasts by secreting TGFß and PDGF, while fibroblasts promote the maturation of Mo_AMs through secreting MCSF [8, 9]. In contrast, while pirfenidone and nintedanib act by suppressing TGFß and PDGF secretion in fibroblasts, these treatments cannot reverse or even stop fibrosis and FVC%pred (forced vital capacity) [2, 10]. FVC, which measures the total amount of gas exhaled from the point of maximal inspiration during the pulmonary ventilation tests, has long been used as a standard and established measurement for evaluating levels of lung fibrosis in interstitial lung disease. The questions arise, therefore, of whether other intercellular communication is involved in the fibrosis process.

Using mult-iomics analysis, we aimed to delineate the effects of Mo_AMs on the development of IPF. Our study revealed the cellular communications between Mo_AMs and fibroblasts and also probes the related mechanisms in vitro. We further identified open chromatin regions and their binding transcription factors (TFs) in Mo_AMs.

## Methods

### Dataset availability

Five single-cell RNA sequencing (scRNAseq) datasets of lung tissue from patients with IPF and control cohorts were obtained from the Gene Expression Omnibus database: GSE 136831, GSE 122960, GSE 135893, GSE 128033, and GSE 132771 [11–15]. Additionally, one scATATCseq dataset was directly extracted from GSE 214085 [16]. Furthermore, we also downloaded the bulk RNA-sequencing dataset GSE 32537, which is the public largest IPF lung tissue bulkseq dataset with the data of lung function test [17]. All patients enrolled in this study were being diagnosed as IPF. Patients with diagnosis of lung cancer, COPD, asthma and other interstitial lung disease were be excluded in this study.

### Data preprocessing and cell doublets removal

Each sample underwent processing using the Seurat package (version 4.11) in RStudio (version 4.12) [18]. The same quality control and normalization were performed on all five datasets. To ensure data quality, cells that expressed less than 300 genes and with over 20% mitochondrial genes were excluded from the analysis. For normalization purposes, gene expressions were scaled by multiplying by a scale factor of 10,000 and subsequently log-transformed. A subset of 3,000 highly variable genes were then selected. Principal component analysis (PCA) and uniform manifold approximation and projection (UMAP) dimensional reduction were applied prior to the removal of doublets. The Doubletfinder package (V3 version) was employed to predict doublets in every sample [19]. To determine an optimal pK value, BCmvn was used, with a PN value set to 0.25 and nExp to 0.08 × nCells^2^/10,000.

### Batch effect correction, unsupervised clustering analysis, dimensional reduction, and cell type annotation

All cohorts were integrated using an orthogonal approach in Seurat, applying the *FindIntegrationAnchors* and *IntergrateData* functions. Following the established workflow of the Anchors integration approach, the integrated data were scaled and PCA analysis was performed. The optimal number of principal components was determined via the *ElbowPlot* function. Clustering was carried out by applying the *FindNeighbors* function with dimensions 1–25 and the *FindClusters* function with resolution 0.1–2. The resulting clusters were then visualized using 2D UMAP. A similar pipeline was applied for the subsequent subclustering analysis. To annotate cell types, differentially expressed genes (DEGs) among different groups were identified using the *FindAllmarkers* function with the Wilcox test approach. The parameters used for this analysis were as follows: only.pos = TRUE, min.pct = 0.2, logfc.threshold = 0.25.

### Trajectory inference and pseudotime analysis

Three methods were employed together to assess the pseudotemporal ordering of Mo_AMs, including the Slingshot package (version 2.40), Monocle 2 and Monocle 3 (version 1.2.9) [20–22]. The top 20 genes that exhibited significant changes during the trajectory were identified with the *graph_test* function and Moran I test in Monocle 3. To calculate the velocity from fibroblasts to myofibroblast, the scVelo package (version 0.2.5) was applied in Python with the dynamic model by importing loom files [23].

### Intercellular communication evaluated with CellChat package

To elucidate the cellular interaction and potential mechanisms between Mo_AMs, epithelial cells, and fibroblasts, we employed the CellChat package (version 1.5.0) [24]. Briefly, the *createCellchat* function was first used to generate a CellChat object. Subsequently, the CellchatDB database, which includes not only ligand-receptor interaction data but also pathway information, was imported for the next analysis. Finally, the functions *computeCommunProbPathway* and *aggregateNet* were used to infer the potential pathways and cellular communication across distinct clusters.

### Score of metabolism, transcription factor activity, tissue repair and phagocytosis

The activity of PPARγ and PU.1 (SPI1) TFs was investigated using the DorothEA database (version 1.8.0), which includes 1,541 human TF-target interactions and uses collated chipseq data to improve accuracy [25]. To compare the metabolic levels among Mo_AMs and TRMs, the scMetablism package was applied (version 0.2.1), which integrates metabolic-related gene sets from both the KEGG and the REACTOME databases [26]. The *sc.metabolism.Seurat* function was used to quantify the metabolism in scMetabolism package. We applied the REACTOME database and selected representative lipid and energetic metabolic pathways which were most associated with the biological function of macrophages. The tissue repair score and phagocytosis score were inferred by AddModuleScore function. And the gene sets were listed in Table_S2.

### Integration of bulkseq and scRNAseq

To determine the clinical subtype of Mo_AMs associated with fibrosis level, the R package Scissor was used [27]. Based on the official tutorial for the *Scissor* function, which involves the Gaussian distribution and Lasso regression for continuous variables, we computed the proportion of Scissor + cells that exhibited a positive correlation with FVC%pred, while Scissor − cells displayed a negative correlation with FVC%pred. The function *reliability.test* was employed to assess the significant differences.

### Cell culture and flow cytometry

**Fig. 1:**
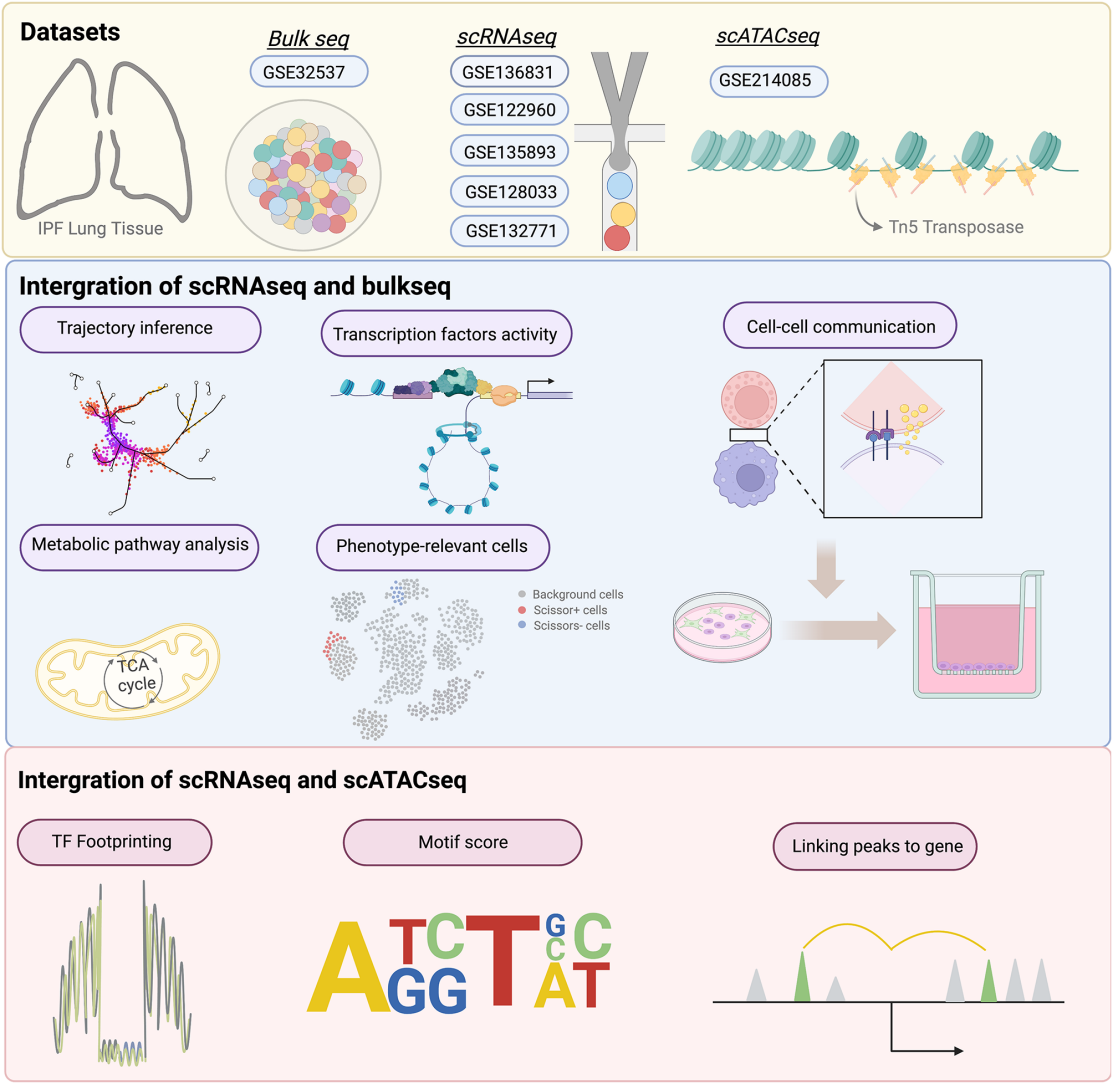
Schematic diagram of the overall study concept

In present study, THP1, the human monocytic leukaemia cell line was used to generate macrophages. HELF, the human embryonic lung fibroblast was applied to build the fibrosis model. Besides, HELF (#CL0676, Fenghui) cells were cultured in DMEM (#12491-015, Gibco) in the presence of 10%FBS (# 10099-141, Gibco). THP1 cells (#CL-0233, Pricella) were grown in RPMI 1640 medium (#PM150110, procell) containing 10% FBS (# 10099-141, Gibco). Passages ranging from 2 to 6 were used for all experimental procedures. To investigate the effect of TGFß on fibroblasts and establish a fibrosis cell model, as previously described, HELF cells were treated with 5 ng/ml TGFß1 (HZ-1011, Proteintech) for a duration of 24 h. To generate anti-inflammatory and profibrotic M2 macrophages, THP1 cells were differentiated into macrophages via priming with 150 nM PMA for 24 h. M2 macrophages were obtained by incubating with IL4 (20 ng/ml) and IL13 (20 ng/ml) for an additional 72 h. To assess the M2 phenotype, the expression of the classic M2 marker CD206 was examined. Briefly, the cells were suspended in the mouse antihuman CD206 antibody for cell labelling (BD Pharmingen, #555,954) and incubated in the dark for 30 min. The conjugated fluorescence was detected using FACScalibur, and the data were analysed using flowJo 10 (version 8.1). For transwell coculture of active fibroblasts and M2 (Fig. 6E), Corning transwell inserts with 0.4 μm pores were used (polycarbonate transwell inserts). M2 (1 × 10^5^) cells were plated in the inner insert, while fibroblasts (1 × 10^5^) were plated in the well bottom. The coculture system was treated with the presence or absence of AMD1300 (#HY-10,046, MCE), pirfenidone (#HY-B0673, MCE), U0126 (#HY-12,031, MCE), PD98059 (#HY-12,028, MCE) and NUCC-390 (#HY-111,793,MCE) for 24 h (See Fig. 1).

### Western blot (WB), quantitative polymerase chain reaction (qPCR), immunofluorescence staining and ELISA

WB experiments were conducted following the previously described procedure. Briefly, for protein extraction, cells were lysed in ice-cold RIPA buffer. The protein concentration was determined using the BCA assay. Protein samples were resolved and separated using 10% SDS-PAGE and subsequently transferred into a PVDF membrane. After blocking with 5% BSA for 1 h, the membranes were subjected to overnight incubation at 4℃ with primary antibodies targeting CXCL12 (Rabbit, #17042-1-AP, 1:3000, proteintech), CXCR4 (Mouse, #60042-1-Ig,1:2000, proteintech), p-MEK (Rabbit,#28930-1-AP,1:2000, proteintech), MEK (Rabbit, #11049-1-AP, 1:10000, proteintech), p-ERK (Rabbit, #28733-1-AP, 1:5000, proteintech), ERK (Rabbit, #51068-1-AP,1:10000, proteintech), α-SMA (Rabbit, #67735-1-Ig, proteintech), and collagen type 1α (Mouse,1:10000,#67288-1-Ig, proteintech), followed by a 1 h incubation with secondary antibodies (Rabbit, #SA00001-2, proteintech; Mouse,# SA00001-2, proteintech). Additionally, to investigate the impact of CXCL12 on the MEK/ERK pathway of M2 macrophages, M2 macrophages were stimulated with the different time points (0 h, 4 h, 8 h) and different concentrations (10 ng/ml and 50 ng/ml) of CXCL12. To further explore the potential role of the CXCX12/CXCR4 axis on the fibrosis model, we cocultured the activated HELF cells with M2 macrophages and M2 macrophages were stimulated with agonist (NUCC-390) and inhibitors of the ERK pathway (U0126 and PD98059). All proteins in the immunoblots were visualized and quantified using ECL. Densitometry analysis was performed in the ImageJ software.

For qPCR, total RNA extraction was carried out using an RNA extraction kit (# G3640-50T, Servicebio). The concentration and purity of the RNA were assessed spectrophotometrically at 260 nm and 280 nm. The cDNA was synthesized using a cDNA synthesis kit (#G3333-50, Servicebio), followed by PCR amplification using SYBR Green PCR master mix (#G3326-01, Servicebio) on the Bio-Rad Real-time PCR instrument. The relative expression was then calculated using the comparative ΔΔCT method. GAPDH or β-actin was utilized a reference gene to normalize relative gene expression levels. The primer sequences were designed using Primer-5 software and synthesized by Invitrogen. The primer pairs can be found in Table S3.

For immunofluorescence staining, cells (1 × 10^5^) were seeded onto glass coverslips. The cells were then fixed with 4% PFA at room temperature for 2 h and gently washed three times with PBS. Cells were incubated with α-SMA (Mouse, #14395-1-AP, 1:500, proteintech) and Collagen type I (Rabbit, 1:400, #67288-1-Ig, proteintech) at room temperature for 1 h. Following multiple washing steps, the cells were labelled with the antirabbit antibody for 30 min at room temperature (Mouse, 1:100,#SA00009-1, proteintech; Rabbit, 1:100,#SA00009-2, proteintech). The fluorescence images were acquired using an epifluorescence microscope (Olympus, BX60).

For ELISA experiments, the supernatants of THP1, M0 macrophages and M2 macrophages were harvested for the measurements of IL10, CCL18 and CCL22 by human ELISA kits (Human IL-10 Elisa Kit, #EH10245S, biotech, shanghai; Human CCL18/PARC Elisa Kit, #EH10070M, biotech, shanghai; Human MCP-1/CCL2 Elisa Kit, #EH10335S, biotech, shanghai) according to the manufacturers’ instructions.

### Interference transfection and transwell migration assays

CXCR4 knockdown was conducted followed by M2 macrophages seeded into 6-well plates at cell densities of 60-70%. M2 macrophages were transfected with siRNA via applying Lipofectamine 3000 (#L3000015, Thermos). The CXCR4 siRNA sequence (GGCAAUGGAUUGGUCAUCCUGGUCA) and the control sequence (GGCGGUUUAUGGUACUCCGGAAUCA). WB analysis was performed to validate the siRNA knockdown.

Transwell migration assays were conducted in 24-well transwell inserts with 8 μm pore size (Corning) (Fig. 6B). To obtain the media from the fibrosis model, HELF cells of logarithmic growth phase cells were seeded into 10 cm dishes and the media were collected until the density was 70–80% confluence. M2 macrophages (5 × 10^5^) were introduced into the upper chamber, while the conditional media from the fibrosis model with or without AMD3100 and CXCR4 siRNA were seeded into the lower chambers. Following incubation of the transwell plates at 37 °C with 5% CO_2_ for 24 h, the migrated cells using crystal violet solution (1%) and visualized through immunofluorescence. Image-Pro Plus software (Media Cybernetics Corporation, USA) was used for counting cell numbers.

### scATATCseq processing and integration with scRNAseq

The scATATCseq analysis was conducted using ArchrR (version 1.02), with five fragments used as input for subsequent processing [28]. Fragments exhibiting transcription start site enrichment below 4 and number of unique fragments (log10) below 3 were excluded. After excluding doublets using the *filterDoublts* function, we employed iterative LSI reduction (*addIterativeLSI* function) to eliminate batch effects. We then aligned the scATATCseq with our scRNAseq, which had been annotated using the *addGeneIntegrationMatrix* function derived from the CCA method in Seurat. Subsequently, we generated pseudobulk replicates to calculate motif enrichment analysis and used MACS to call peaks. Following this, we compared comparisons of peaks and TFs between Mo_AMs and TRMs. The *AddPeak2GeneLinks* function was invoked to determine the regulatory association between peak accessibility and gene expression. For the Mo_AMs analysis, we constructed a differentiation trajectory similar to the scRNA analysis using the *addtrajectory* function. Lastly, to identify TF drivers along the trajectory, we employed the *correlateTrajectories* function to integrate gene expression and motif accessibility. These regulatory drivers were visualized through heatmaps.

### Statistical analyses

Statistical analysis was conducted by Graphad Prism 9 and R (version 4.2.0) via t test or one-way ANOVA. *P* < 0.05 was considered significant (*p < 0.05; **0.05 < *p* < 0.01; ***p < 0.001).

## Results

### Mapping scRNAseq atlas of multiple lung cell types in idiopathic pulmonary fibrosis

**Fig. 2:**
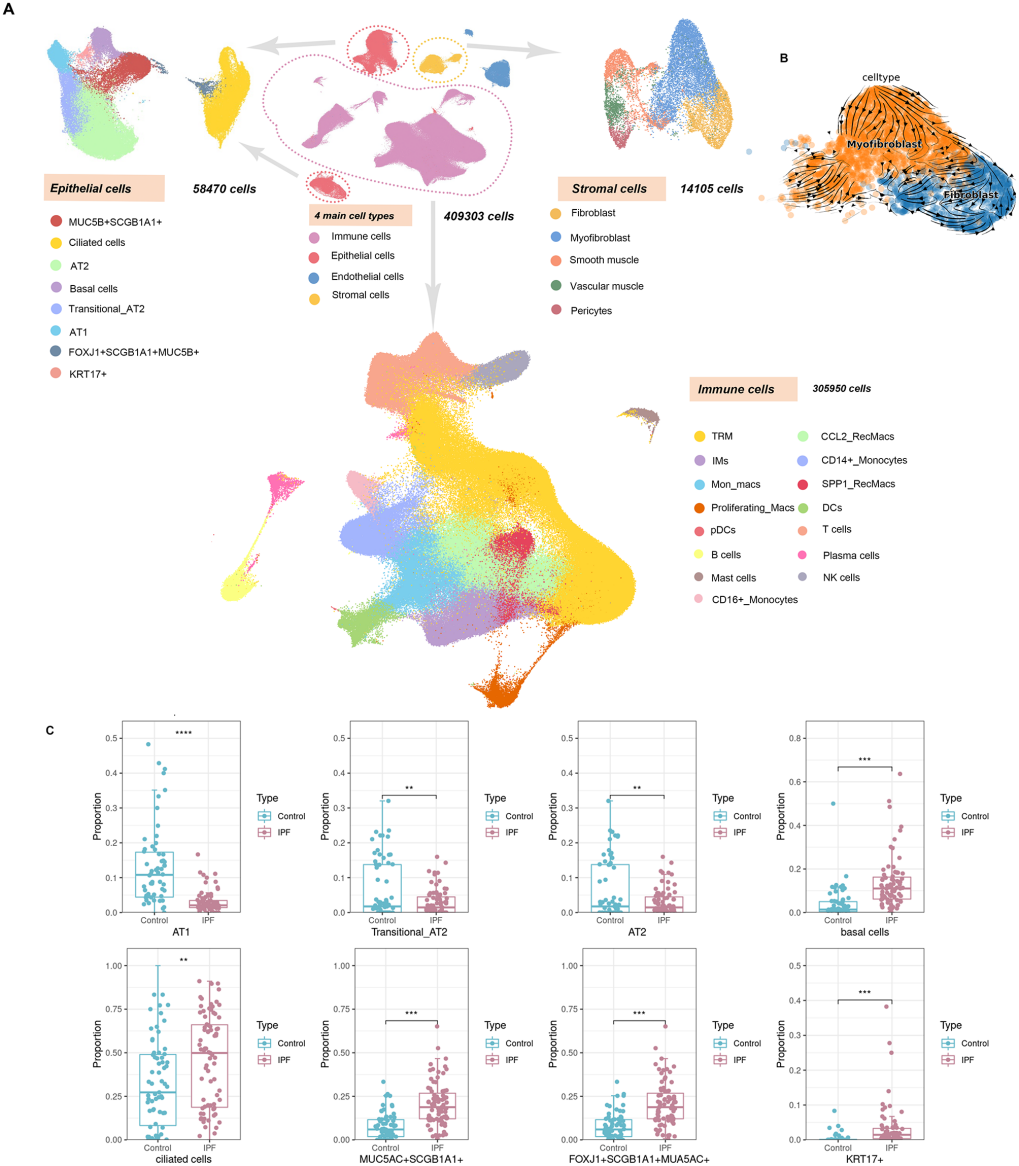
Overview of the Idiopathic Pulmonary Fibrosis (IPF) Atlas. The UMAP plots of epithelial cells, stromal cells, and immune cells (**A**). Trajectory of fibroblasts and myofibroblasts using scVelo (**B**). Proportion of epithelial clusters in IPF group and control group (**C**)

The demographic and clinical characteristics of the scRNAseq and scATATCseq samples are listed in Table S1. To mitigate batch effects, an anchor-based strategy was employed and no significant batch effects were observed (Fig. S1A–D). After rigorous filtering and quality control, our scRNAseq atlas contained 409,303 cells, including 58,470 epithelial cells, 14,105 stromal cells, and 305,950 immune cells (Fig. 2A). The expression levels of marker genes, which were used for cell type annotation and identification of cell clusters, are depicted in the feature plots and dotplots, aligning with existing knowledge (Fig. S2A, C–E). In accordance with the prevailing understanding of the aetiology of IPF, our findings demonstrated fewer alveolar epithelial cells in the IPF group compared to the control group (Fig. 2C). Conversely, the IPF patients exhibit an increase in epithelial secretory cells and ciliated cells, which may contribute to enhanced elimination of environmental risk factors and the promotion of fibrosis development. Additionally, the IPF group displayed a higher abundance of stromal cells than the control group (Fig. S2B). Furthermore, a phenotypic transition from fibroblasts to myofibroblasts was also evident in the IPF patients (Fig. 2B).

### SPP1_RecMacs originate from CD14+_Monocytes rather than tissue-resident alveolar macrophages

**Fig. 3:**
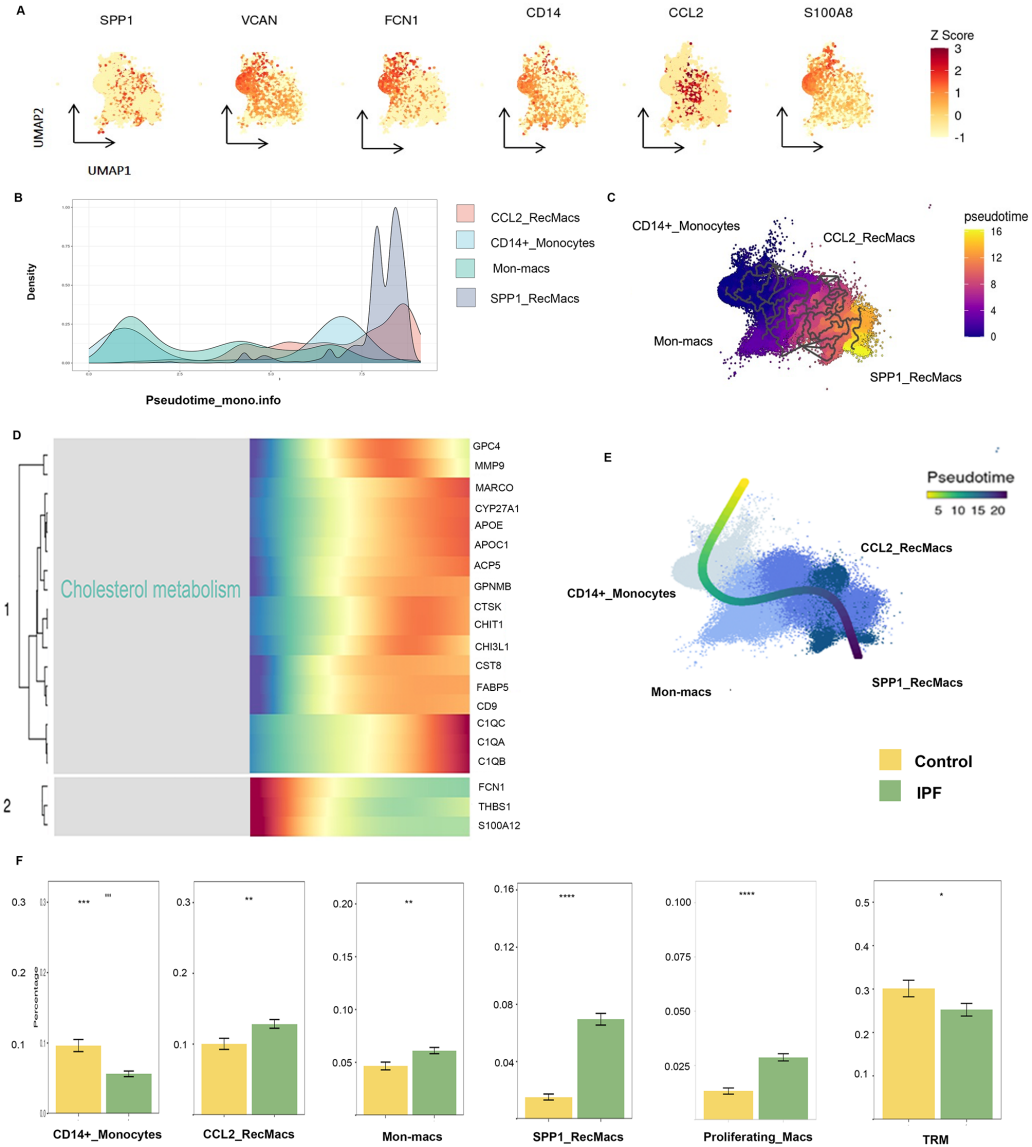
SPP1_RecMacs Derive From Monocytes. Maker gene expression in monocyte-derived alveolar macrophages (Mo_AMs) projected onto UMAP (**A**). Monocle (**B, C**) and Slingshot (**E**) trajectories showing the progression of CD14_Monocytes, Mon-macs, CCL2_RecMacs, and SPP1_RecMacs. The gradient colors reflect the changes along the trajectory (**C, E**). Heatmap of top 20 gene expression changes along the Monocle trajectory (**D**). Proportion of Mo_AMs in the idiopathic pulmonary fibrosis group and the control group (**F**)

Fifteen distinct immune cell types were identified, as shown in Fig. 2A. Among these, we identified eight monocyte and macrophage clusters, including CD14+_Monocytes (*CD14, FCN1, S100A8, S100A12, VCAN, ILIR2*), CD16+_Monocytes (FCGR3A, LILRA5, APOBEC3A, MTSS1), Mon_macs (*CLEC10A1, GPR183, FCGR2B, CD93, SDS*), CCL2_RecMacs (RecMacs, recruited macrophages;*CCL2, EMP1, RNASE1, MARCO, MRC1, MSR1*), interstitial macrophages (*LGMN, FOLR2, PLTP, CCL13*), SPP1_RecMacs (*SPP1, TREM2, CHI3L1, MMP9, PLA2G7, CHIT1*), proliferating_Macs (*MKI67, TOP2A, UBE2C, CDK1, BIRC5*), and TRMs (*FABP4, INHBA, SERPING1, MME, RBP4*). Interestingly, a decreased proportion of CD14+_Monocytes was observed, while a trend was noted towards an increase in Mon-macs, CCL2_RecMacs, and SPP1_RecMacs (Fig. 3F). Compared to Mon-macs and CCL2_RecMacs, SPP1_RecMacs exhibited a higher level of *CHIT1*, *SPP1*, *SDC2*, and *CHI3L1* (Fig. 3A, Fig. S2E). *CHIT1*, a well-known chitinase, was implicated in various inflammatory lung and fibrotic diseases [29–31]. *CHI3L1* was also found to contribute to the development of lung fibrosis [33]. Notably, *SDC2*, encoding a glycosylated integral membrane protein, was discovered to alleviate fibrosis by decreasing TGFß1 receptors in epithelial cells [32].

Considering the crucial role of Mo_AMs in the development of IPF and the unclear origin of SPP1_RecMacs, we conducted Slingshot and Monocle 2/3 analysis to capture a linear pseudotime process starting from CD14+_Monocytes and progressing towards Mon-macs, CCL2_RecMacs, and SPP1_RecMacs as the trajectory endpoint (Fig. 3B–C, E). Among the top 20 variable genes associated with this trajectory, *APOE* exhibited an increase, which was in line with previous research findings [33] (Fig. 3D). Furthermore, our analysis revealed that the upregulated genes within the trajectory exhibited enrichment in the “cholesterol metabolism” KEGG term (Fig. 3D). The TF analysis revealed that both PU.1 and PPARγ tend to be expressed higher in SPP1_RecMacs than in TRMs (Fig. S3A, B). Moreover, SPP1_RecMacs also displayed a significantly lower phagocytosis score and higher tissue repair score than TRMs (Fig. S3C–D, Table S2). Lastly, SPP1_RecMacs exhibited greater metabolic activities than TRMs in IPF patients, including lipid and phospholipid metabolism, glucose metabolism, and energy metabolism (Fig. S3E).

### Identify the Mo_AMs subtype associated with FVC%pred

**Fig. 4:**
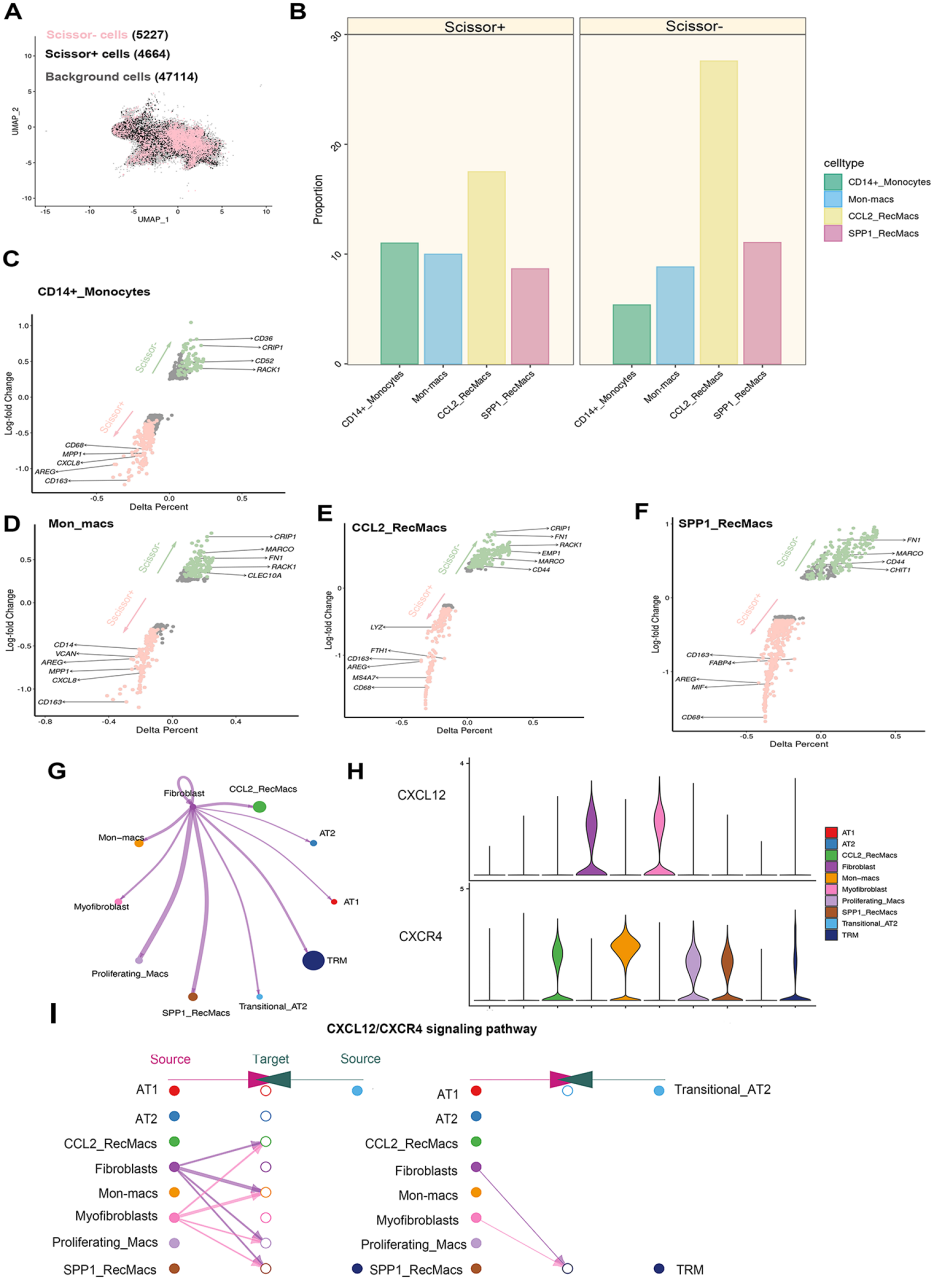
Phenotypic Mo_AMs Subpopulations and Cellular Interaction Between Fibroblasts and Mo_AMs. UMAP plots identify the cells with the higher levels of FVC%pred (Scissor+) and the cells with lower levels of FVC%pred (Scissor−; **A**). Barplots show the constitution of Mo_AMs in Scissor + cells and Scissor − cells (**B**). Volcano plots of differentially expressed genes in Scissor + cells versus Scissor − cells in Mo_AMs (**C–F**). The ligand-receptor analysis with CellChat (**G**). The expression of CXCR4 and CXCL12 (**H**) and the hierarchical plot (**I**) of the CXCL12/CXCR4 signalling pathway

In this study, we analysed 119 IPF bulk samples with FVC%pred information and used the Scissor package to identify fibrosis-related Mo_AMs among a total of 57,005 cells. Of these cells, 5,227 Scissor − cells were negative for FVC%pred, and 4,664 Scissor + cells were positive for FVC%pred (Fig. 4A). As previously indicated, Scissor − cells predominantly occupy the latter portion of the trajectory, specifically CCL2_RecMacs and SPP1_RecMacs, suggesting the crucial role of Mo_AMs in promoting fibrosis (Fig. 4B). This result is further enhanced by the statistically significant *p*-value of the reliability significance test (*p* < 0 0.001). To shed light on the transcriptional differences of Scissor + and Scissor − cells, we compared their DEGs. Our analysis revealed an upregulation of macrophage markers (*MARCO*, *FN1*, *EMP1*, and *CHIT1*) in Scissor − cells, while macrophage markers (*AREG*, *CD163, FABP4*, and *LYZ*) were found to be greater in Scissor + cells (Fig. 4C–F). Among them, FN1 could promote fibrosis via encoding fibronectin [56]. CHIT1, as classic maker for activating macrophages, was found to be highly expressed in IPF patients and can be used as therapeutic targets [57, 58]. AGER can foster inflammation by secreting cytokines such as IL-6, IL-8 and GM-CSF [59]. FABP4 could also regulate lipid metabolism by PPARγ and facilitate inflammation [60].

### Fibroblasts induce chemotaxis of Mo_AMs by CXCL12/CXCR4 axis in idiopathic pulmonary fibrosis

**Fig. 5:**
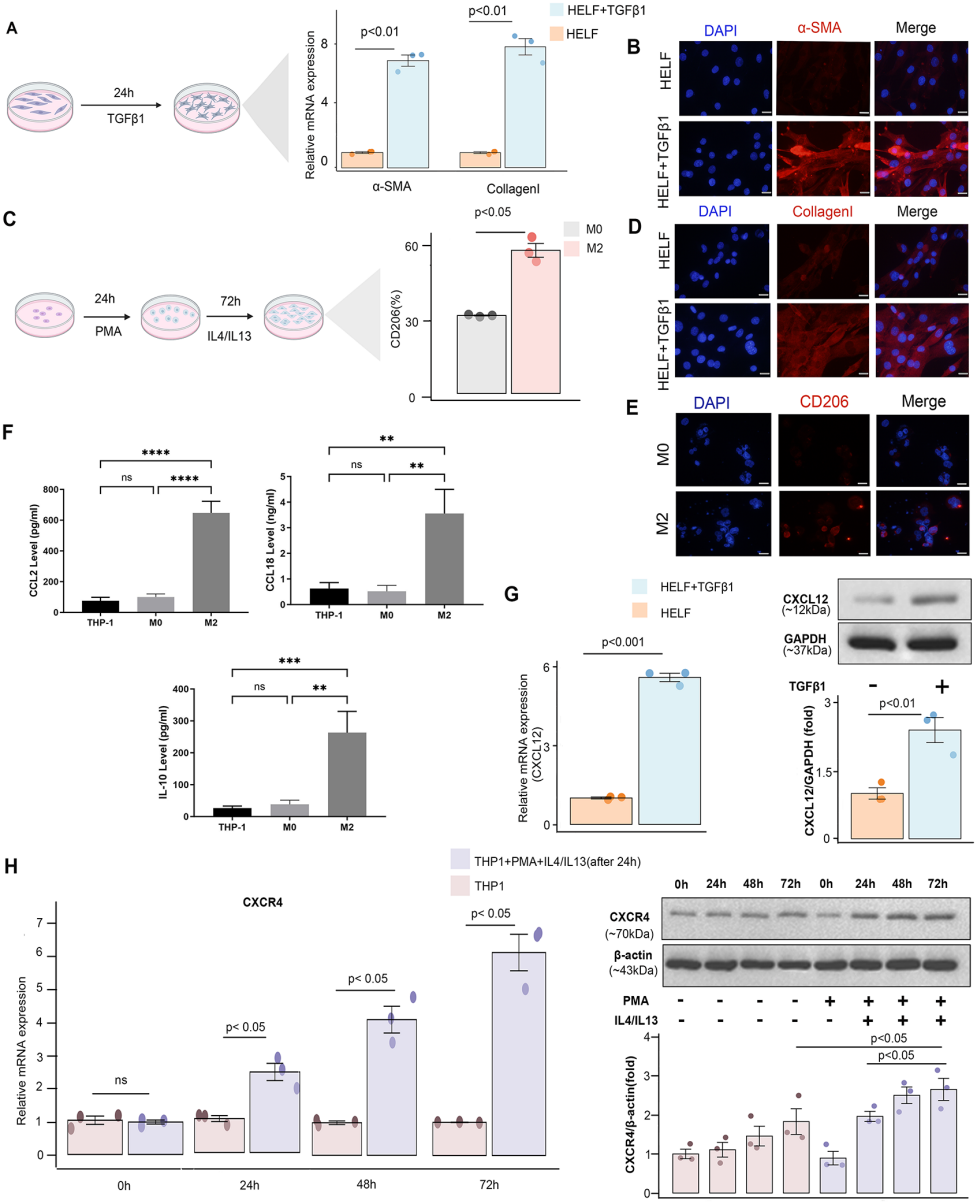
High Expression of CXCR4 in Activated Fibroblasts and High Expression of CXCL12 in Profibrotic Macrophages. Establishment of the fibrosis model and expression validation of *α*-SMA and collagen type I expression by quantitative polymerase chain reaction (qPCR) and immunofluorescence staining (**A, B, D**; scale bars = 20 μm). Inducing M2 macrophage formation and validation of CD206 expression via flow cytometry (**C, E**). Validation of CCL2, IL10 and CCL18 in M2 macrophages by ELISA (**F**). RNA and protein expression of CXCL12 in unstimulated HELF cells and stimulated HELF cells through qPCR and Western blot (**G**). RNA and protein expression of CXCR4 in unstimulated THP1 cells and stimulated THP1 cells through qPCR and Western blot (**H**)

Intercellular communication among epithelial cells, myofibroblasts, fibroblasts, and alveolar macrophages was observed in patients with IPF. The use of CellChat revealed the CXCL12/CXCR4 signalling pathway between Mo_AMs and fibroblasts as well as myofibroblasts (Fig. 4G, I). The reliability of the communication was confirmed by the expression of CXCL12 and CXCR4 (Fig. 4H). To further validate these findings, coculture experiments of the fibrosis models and Mo_AMs were performed. To establish the fibrosis model, the HELF cell line was selected and was induced with TGFβ1, followed by assessment of the expression of α-SMA and collagen type I as the markers of fibrosis. The mRNA expression of α-SMA and collagen type I was found to be upregulated in the model (Fig. 5A). The results were also further confirmed by immunofluorescence analysis (Fig. 5B, D). We followed established protocols to induce polarization THP1 cells into M2 macrophages and found that the cell surface marker CD206 was significantly upregulated in M2 macrophages compared to M0 macrophages (Fig. 5C, E) [34]. ELISA results showed that the protein levels of IL10, CCL2 and CCL18 were highly expressed in M2 macrophages (Fig. 5F). Additionally, CXCL12 was observed to increase in HELF cells upon TGFβ1 induction, whereas CXCR4 was found to be increased during M2 polarization (Fig. 5G, H).

**Fig. 6:**
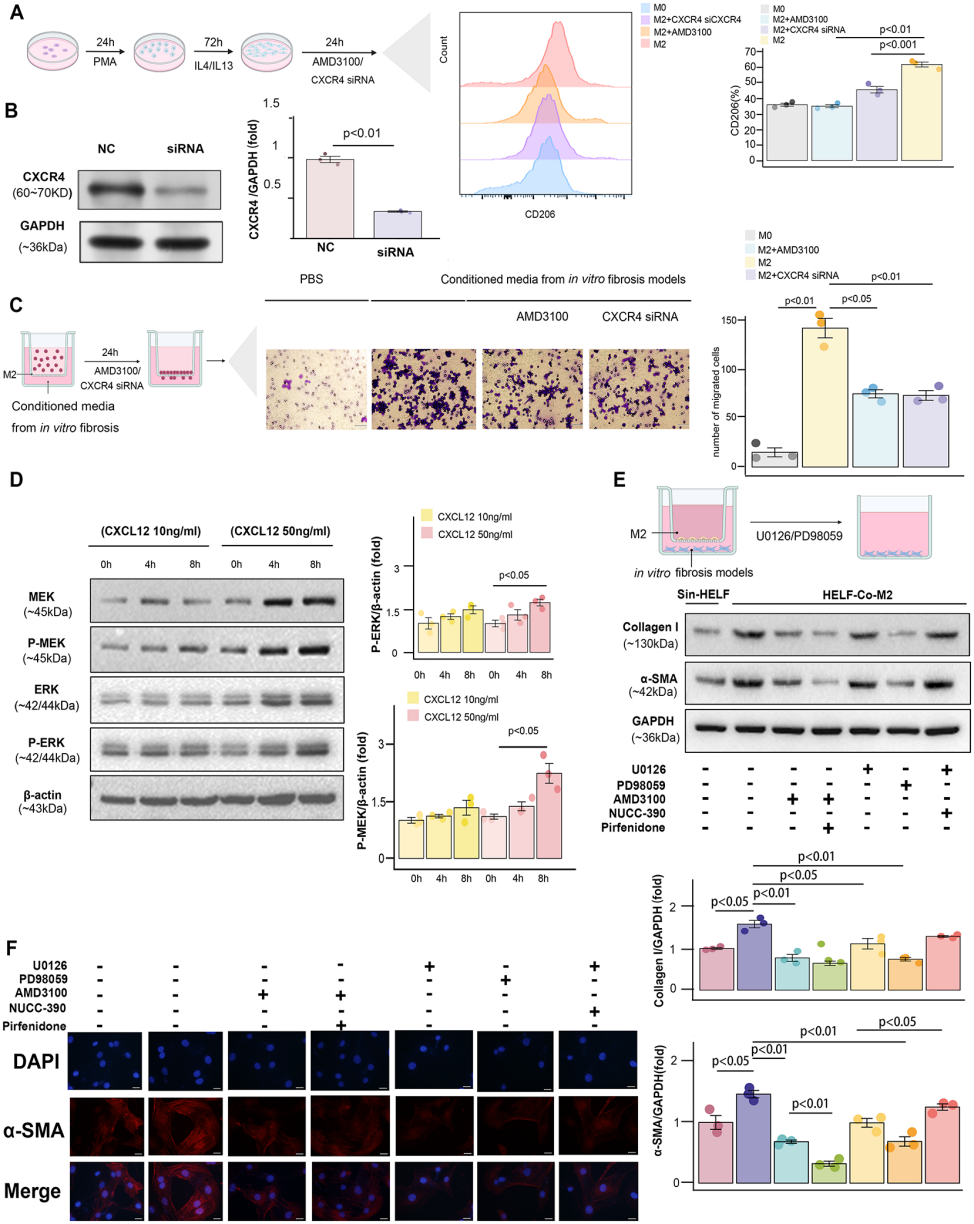
The CXCL12/CXCR4 Pathway Can Efficiently Stimulate the Chemotaxis of M2 Macrophages, Promoting the Activation of Fibroblasts. The expression of CD206 measured by M2 macrophages with the stimulation of AMD3100 (CXCR4 antagonist) and CXCR4 siRNA for 24 h (**A**). The efficacy of siRNA CXCR4 was verified by WB (**B**). Bar graph represents changes of protein expression on CXCR4 (**B**). M2 macrophages were cultured in conditioned media from the fibrosis models with or without AMD3100 and CXCR4 siRNA. M2 macrophages were seeded into the upper chambers of transwell. The conditioned media from in vitro fibrosis models was added into the lower chambers of transwell (**C**). Western blot showing expression of MEK, P-MEK, ERK, and P-ERK in M2 macrophages at different time points (0 h, 4 h, 8 h) and different concentrations (10 ng/ml and 50 ng/ml) of CXCL12 stimulation (**D**). *Bar* graph represents changes of protein expression on pERK and pMEK (**D**).Western blot of α-SMA and collagen type I and immunofluorescence of α-SMA in activated HELF cells cocultured with M2 macrophages and then stimulated with agonist (NUCC-390) and inhibitors of the ERK pathway (U0126 and PD98059), CXCR4 inhibitor (AMD3100), and antifibrotic agents (pirfenidone; **E, F**; scale bars = 20 μm). Bar graphs represent changes of protein expression on α-SMA and Collagen I (**E**). M2 macrophages were seeded into the upper chambers of transwell. The fibrosis models were added into the lower chambers of transwell

To further determine the direct involvement of CXCR4 in M2 macrophages, M2 macrophages were then cultured in media from TGFβ1-treated HELF cells, either with or without CXCR4 antagonist (AMD3100) or CXCR4 small interfering (siRNA). siRNA knockdown decreased the expression level of CXCR4 in M2 m macrophages (Fig. 6B). The results demonstrated that inhibition of CXCR4 led to a significant reduction in M2 polarization (Fig. 6A). To investigate the necessity of the CXCL12/CXCR4 axis for M2 chemotaxis to fibroblasts, we collected the supernatant of TGFβ1-treated HELF and used it as chemoattractant for M2 in vitro. The outcomes from the transwell assays revealed that the presence of chemokines in the media derived from the fibrosis model can enhance the chemotactic capacity of M2 macrophages, and this migratory response can be suppressed when AMD3100 and CXCR4 siRNA are used (Fig. 6C). To further explore the underlying mechanisms responsible for chemotactic effects, M2 macrophages were introduced at different time points and concentrations of CXCL12, and the involvement of the ERK pathway was evaluated by WB analysis. We found the expression level of pERK and pMEK increased in a time- and dose-dependent manner (Fig. 6D). To further elucidate the potential role of the CXCX12/CXCR4 axis in M2 macrophages, fibroblasts were then cocultured with M2 macrophages using transwell plates in vitro. The expression of α-SMA and collagen type I was significantly downregulated upon treatment with the ERK pathway inhibitors (PD98059 and U0126). This trend, as anticipated, could be reversed by the CXCR4 agonists (NUCC-390) (Fig. 6E, F).

### Importance of APOE for the differentiation of SPP1_RecMacs

**Fig. 7:**
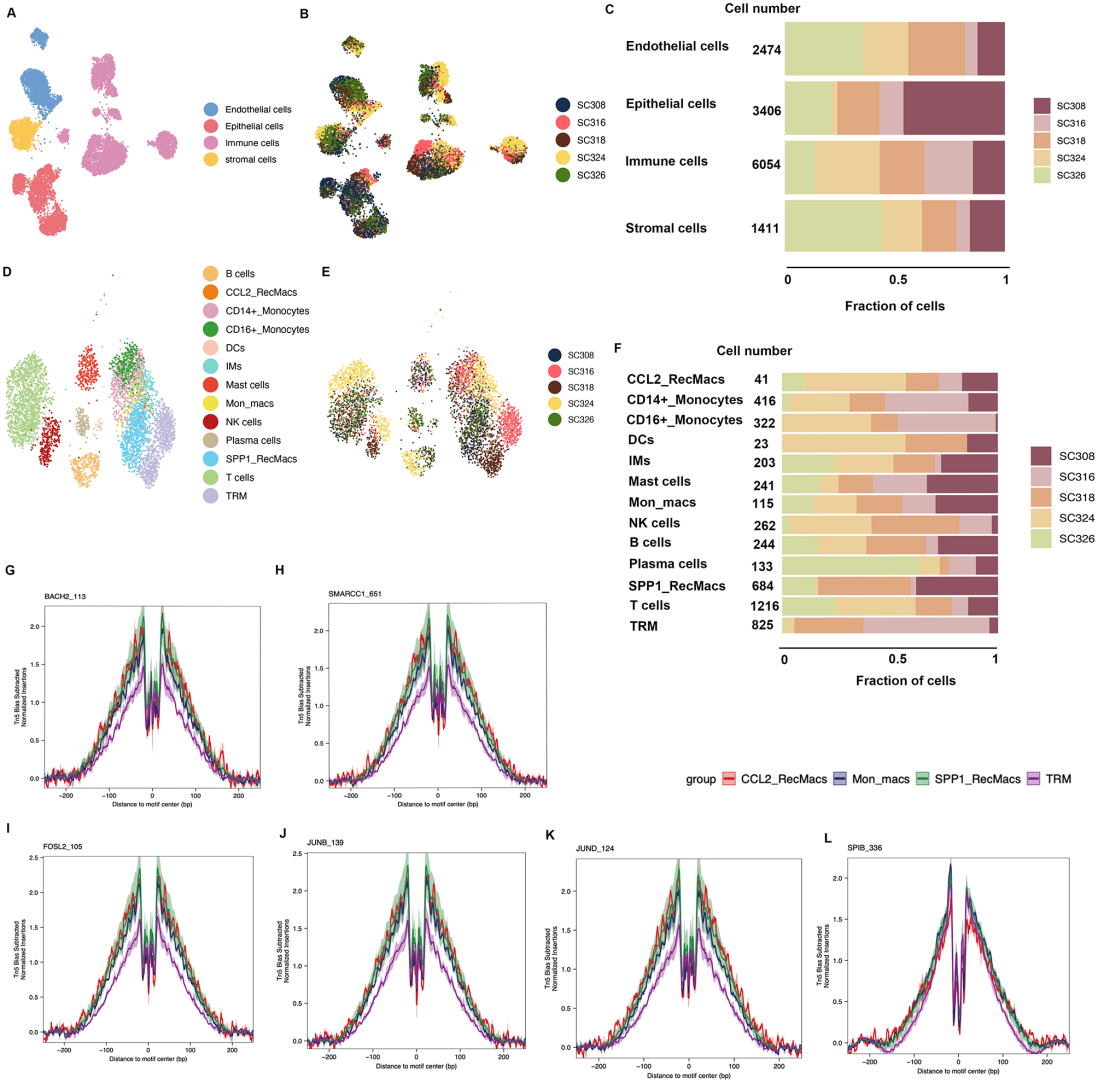
Footprinting Analysis of Transcription factors in Mo_AMs and TRMs. The UMAPs of four main cell types and immune cells annotated via the scRNAseq atlas were coloured by cluster and sample origin (**A, B, D, E**). The stacked barplots illustrate numbers and proportions of each patient in scATACseq (**C, F**). Footprinting analysis of *BACH2*, *SMARCC1*, *FOSL2*, *JUNB*, *JUND* and *SPIB* in monocyte-derived alveolar macrophages and tissue-resident alveolar macrophages (**G–L**)

**Fig. 8:**
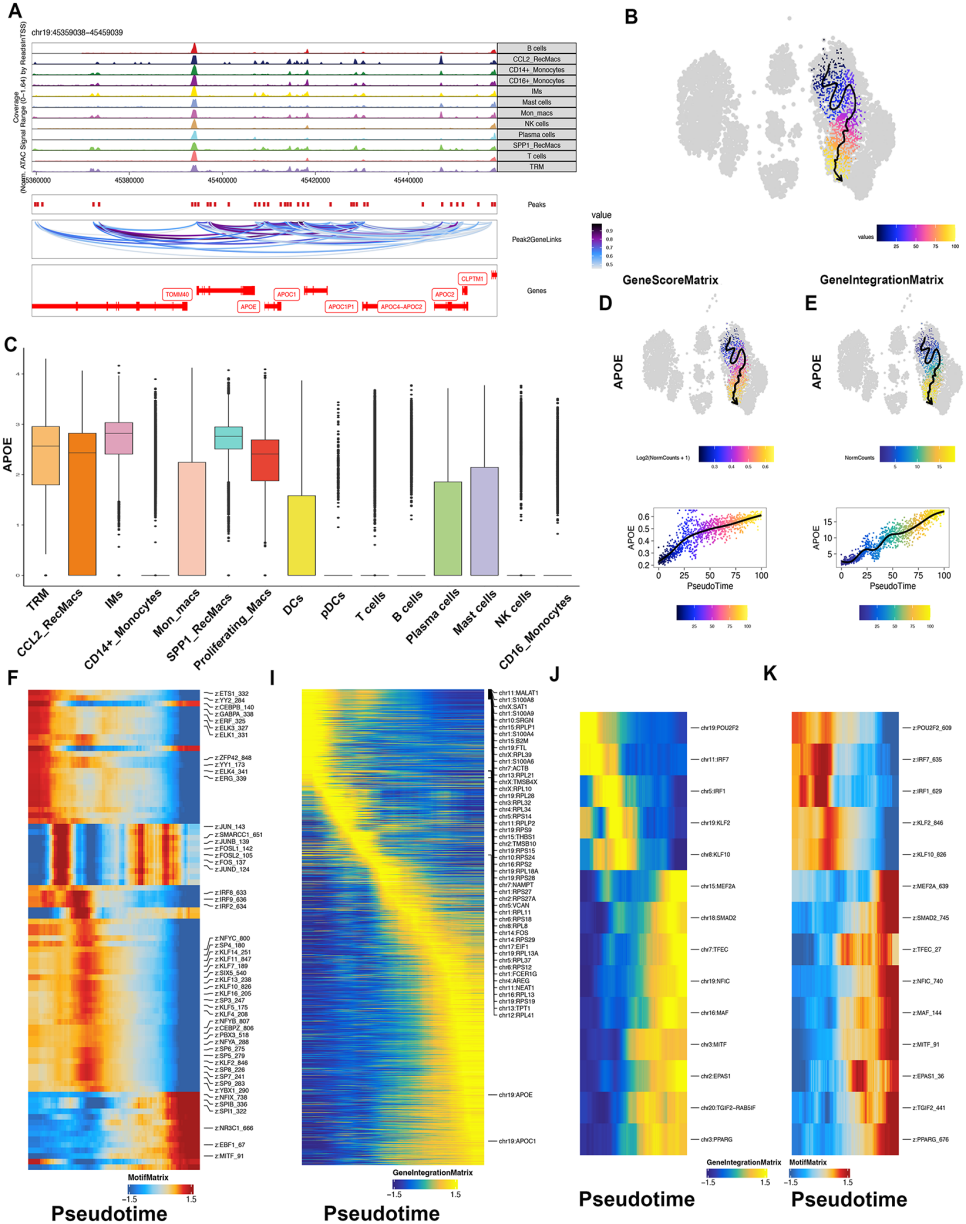
Identification of the Regulatory Drivers in Mo_AMs. Browser track showing peak_gene_link at the APOE locus in all immune cell types (**A**). The expression of APOE in the scRNAseq atlas is shown for all immune cell types (**C**). Inferred differential trajectory of Mo_AMs via ArchR (**B**). Activity of APOE during the Mo_AMs trajectory via the GeneScoreMatrix and GeneIntegrationMatrix (**B, D, E**). Heatmap along the pseudotime trajectory via the MotifMatrix and GeneIntegrationMatrix (**F, I**). Identification of transcription factor drivers through integration analysis of the MotifMatrix and GeneIntegrationMatrix (**J, K**)

Next, to trace the regulatory mechanisms associated with transcriptional processing for Mo_AMs, we projected the scATATCseq data (GSE214085) with our scRNAseq atlas. Following stringent quality filtering and exclusion of doublets in the scATACseq dataset, 13,345 cell profiles, specifically 6,054 immune cell profiles, were obtained using ArchR (Fig. 7C, S4). Moreover, no discernible batch effects were observed upon LSI integration (Fig. 7A, B,D, E). Through the CCA integration method, the cluster annotation of scATACseq can be accurately labelled consistently with our scRNAseq cell types. Within the samples, SC308, SC318, and SC326 belong to the IPF group. SC316 and SC324 belong to the control group. The total cell numbers of each sample are listed as follows: 2988 (SC308), 1941 (SC316), 2736 (SC318), 2623 (SC324) and 3057 (SC326). Besides, the cell numbers of SPP1_RecMacs are: 268(SC308), 23(SC316), 186 (SC318), 29(SC324) and 178(SC326). Thus, despite the presence of potential heterogeneity among the samples, the IPF group tended to exhibit a higher proportion of SPP1_RecMacs compared to the control group (Fig. 7C, F). The foot printing analysis, which mirrored and assessed the genome binding sites of TFs, revealed that *JUNB*, *BACH2*, *FOSL2*, *JUND*, *SPIB (PU.1)*, and *SMARCC1* in Mo_AMs exhibited stronger binding to the wide genome than TRMs (Fig. 7F–L). Notably, APOE is a risk biological factor of lipid metabolisms that affect various fibrosis diseases [33, 47, 48]. Also, APOE was found to be upregulated in alveolar macrophages in patients with IPF and played an important role in Collagen I phagocytosis [33, 55]. In this study, we have noticed deep chromatin accessibility and high expression of APOE in SPP1_RecMacs as well as TRMs (Fig. 8A, C). Through the imputed pseudotime analysis of the scATACseq data, we also successfully illustrated the upregulation of APOE expression in both the GeneScoreMatrix and the GeneIntegrationMatrix during the differential process of Mo_AMs (Fig. 8B–E), which was consistent with trajectory heatmaps in our scRNAseq data and scATATCseq data (Figs. 3D and 8I). Here, it is essential to underscore the differences among the three commonly used matrices (GeneScoreMatrix, GeneIntergrationMatrix and MotifMatrix) in the ArchR framework. The GeneScoreMatrix means the gene activity score inferred by chromatin accessibility data. The GeneIntergrationMatrix integrates the information of scRNAseq and scATACseq, thereby capturing the relationship between chromatin accessibility and gene expression patterns. The MotifMatrix serves as a repository for transcription factor binding site information. Thus, in contrast to conventional scRNAseq trajectory analysis, scATAC-seq pseudotime analysis prioritizes the examination of gene regulation and chromatin state dynamics throughout the process of cell development and differentiation, thereby facilitating the identification of crucial regulatory elements and TFs. Additionally, the motif heatmap also showed a change in chromatin accessibility of *JUNB*, *JUND*, *FOSL2*, *SPIB (PU.1)*, and *SMARCC1* during the trajectory, in line with our results from the analysis of the foot printing and TF activities of our scRNAseq atlas (Fig. 7H and L, S3A). Using a combination of motif matrix and GeneIntegrationMatrix, we ultimately identified that SMAD2 and PPARγ could be the potential drivers during the Mo_AMs differentiation (Fig. 8J–K).

## Discussion

In this study, we provided a multiomics framework analysis of lung tissue from patients with IPF to enable the generation of a large-scale dataset. Although scRNAseq can effectively identify comprehensive gene expression profiles in various cell types, it falls short in providing informative descriptions of clinically relevant data and thus fails to establish associations between these cell types and clinical phenotypes. Moreover, the absence of available epigenetics data hinders our understanding of the upstream mechanisms governing transcriptional regulation in the cell types of interest. To address these limitations, we drew a comprehensive map by integrating scRNAseq with bulkseq and chromatin accessibility data. Mo_AMs played a deteriorative role in the promotion of IPF through various mechanisms, involving intercellular communication between fibroblasts and epithelial cells, metabolic reprogramming, and alteration of phagocytic function [35–37]. This work comprehensively investigated the evidence of the communication between Mo_AMs and fibroblasts, determined the TFs involved in this process, highlighted the significant changes in transcriptional regulation between Mo_AMs and TRMs, and identified clinical-associated Mo_AMs.

Osteopontin (SPP1), a multifunctional glycoprotein, is implicated in various cell types and supports the recruitment and proliferation of monocytes [12]. The current consensus suggests that SPP1_RecMacs are a subset of macrophages in IPF and even in the fibroproliferative phase of severe COVID-19 [38]. Moreover, the knockdown of spp1 in bleomycin-induced mice was found to attenuate lung fibrosis [39]. Moreover, in this study, we also found that SPP1_RecMacs have distinct roles in the functions of phagocytosis, tissue repair, and metabolism. We also provided evidence that SPP1_RecMacs arise from monocytes rather than TRMs. Thus, it is reasonable to surmise that the enrolment of monocytes to the lung tissue could be attributed to the reason that the higher levels of repair mechanisms were required for IPF patients. In light of all our observations, we are tempted to further ask whether SPP1_RecMacs could impair lung function. For the first time, our study revealed a higher proportion of SPP1_RecMacs in the low-FVC subgroup compared to the high-FVC subgroup, which further confirmed that SPP1_RecMacs contribute to the promotion of lung fibrosis. Overall, SPP1_RecMacs could serve as a valuable tool for clinicians in assessing the ratings of IPF.

The CXCL12/CXCR4 axis was considered a key contributor to multiple organ fibrosis such as lung, liver, and heart [40–43]. Additionally, it has been reported that the increased expression of CXCR4 is related to higher mortality in patients with IPF. CXCR4, a prototypical G-protein coupled receptor consisting of 352 amino acids, has been found abundantly expressed in monocytes and serves as a crucial chemokine receptor in fibroblasts [44]. Although recent studies on IPF have confirmed that the high expression of cxcl12 in fibroblastic foci and high expression of cxcr4 in myeloid cells [45, 46], the potential downstream mechanism of CXCR4 in Mo_AMs is still unclear. Herein, we aimed to validate the intercellular communication of CXCR4/CXCL12 pathway between Mo_AMs and fibroblasts. Moreover, we further demonstrated that the binding fibroblasts through cxcr4 leads to the activation of the MEK/ERK pathway in Mo_AMs. Consistent with our hypothesis, blocking of the MEK/ERK pathway in Mo_AMs resulted in the elimination of collagen and α-SMA in the vitro fibrosis model. Previous studies found that MEK1/2 played essential roles in macrophages reparative properties [63]. Additionally, the MEK/ERK pathway could enhance the phagocytosis of macrophages [64]. Clinical trials have also confirmed that the inhibitors of the ERK signaling pathways (hydroxy- chloroquine) could alleviate pulmonary fibrosis [61, 62]. Taking our findings together, we speculate that the inhibiting of the MEK/ERK pathway in Mo_AMs could be effective and promising treatment regimens for patients with IPF.

Next, we aimed to elucidate the regulatory mechanisms governing the differentiation of Mo_AMs and determine the major drivers in the differentiation of Mo_AMs. Our results indicated that the expression of APOE upregulated during the differentiation, which was previously demonstrated by the bleomycin-induced mice [33]. Moreover, the deletion of APOE in Mo_AMs in mice led to the attenuation of atherosclerosis, liver fibrosis, and lung fibrosis [33, 47, 48]. Previous investigations also found that APOE was highly expressed in Mo_AMs [47, 49]. Our study, however, provided evidence supporting the increased expression and chromatin accessibility of APOE not only in SPP1_RecMacs, but also in TRMs. APOE is an apolipoprotein that plays a leading role in lipid metabolism [50]. Thus, this raises the question of why the antifibrotic gene APOE is highly expressed in Mo_AMs. Two potential explanations may be considered: Firstly, Mo_AMs may contribute to lung fibrosis more significantly than any anti-fibrotic benefits provided by APOE. Secondly, the involvement of APOE in collagen engulfment is dependent on the LRP1 receptor, which may not be optimally functioning during early stages of fibrosis [33]. Similarly, although the antifibrotic gene SDC2 is also found to be overexpressed in Mo_AMs, it is suggested that Mo_AMs may also promote lung fibrosis more effectively than the anti-fibrotic effects associated with SDC2. Our metabolitic analysis also revealed that TRMs have a relatively high level of lipid metabolism. Furthermore, we determined that SMAD2 and PPARγ are the potential drivers in the differentiation of Mo_AMs. Firstly, compared with other TFs in Fig. 8J, PPARγ and SMAD2 are the most studied TFs in macrophages. Besides, PPARγ, an important nuclear hormone receptor, modulates lipid metabolism, relates to surfactant turnover and influences AM functionality [51, 52]. Unlike Mo_AMs in a healthy state, we found that SMAD2 could also be key Mo_AMs transcription factors in IPF patients. SMAD2, a member downstream molecule of the TGFβ pathway, is involved in promoting the expression of MHC molecules and regulating phagocytosis in macrophages and thus could be recognized as a major mediator of fibrosis [53, 54]. Thus, the extent to which SMAD2 of Mo_AMs is involved in IPF remains largely unknown and calls for further experimental exploration. However, it is essential to recognize the importance of other TFs identified in Fig. 8J-K, which could also be the focus of research in the future.

We are aware, however, that multiomics analysis may not fully elucidate potential mechanisms, and there are still some shortcomings in this study. Thus, it would be interesting to additionally study the neighbourhood of fibroblasts and macrophages via spatial transcriptomics involved in the cxcl12/cxcr4 pathway. Moreover, we have to acknowledge that there were high variations among individuals. The large-scale IPF atlas would be performed to favour our view. Further investigation is warranted to expand the sample size of the multi-omics analysis in IPF studies, particularly for the scATATCseq analysis.

## Conclusion

Using multiomics analysis, we demonstrated that Mo_AMs are a key factor in promoting the progression of IPF via interacting with fibroblasts. We also identified important TFs which affected Mo_AMs differentiation in patients with IPF.

## Electronic supplementary material

Below is the link to the electronic supplementary material.

Supplementary Material 1

Supplementary Material 2

## Data Availability

Datasets used in this study are available at the Gene Expression Omnibus: GSE 136831, GSE 122960, GSE 135893, GSE 128033, GSE 132771, GSE 214085, and GSE 32537.
