# Correction to: Multiomic analysis of monocyte-derived alveolar macrophages in idiopathic pulmonary fibrosis

**DOI:** 10.1186/s12967-024-05914-0

**Published:** 2024-12-04

**Authors:** Miaomiao Zhang, Jinghao Zhang, Haisheng Hu, Yuan Zhou, ZhiWei Lin, Hui Jing, Baoqing Sun

**Affiliations:** 1 Department of Clinical Laboratory, National Center for Respiratory Medicine, National Clinical Research Center for Respiratory Disease, State Key Laboratory of Respiratory Disease, Guangzhou Institute of Respiratory Health, The First Affiliated Hospital of Guangzhou Medical University, Guangzhou, China; 2 Department of Internal Medicine II, University Hospital Bonn, Section of Pneumology, Bonn, Germany; 3 Department of Respiratory and Critical Care Medicine, Xuzhou Central Hospital, Xuzhou, China; 4 Department of Medicine II, Heart Center Bonn, University Hospital Bonn, Bonn, Germany; 5 Guangzhou Laboratory, Guangzhou, 510005 China

**Correction to: Journal of Translational Medicine (2024) 22:598**

10.1186/s12967-024-05398-y

Following publication of the original article [1], we have been notified that Supplementary Material 2 contained incorrect information.

The original article was updated.

## Electronic supplementary material

Below is the link to the electronic supplementary material.

Supplementary Material 2

## References

[1] Zhang et al. (2024) Multiomic analysis of monocyte-derived alveolar macrophages in idiopathic pulmonary fibrosis (2024). 22:598 10.1186/s12967-024-05398-y 10.1186/s12967-024-05398-y PMC11209973 38937806

