# Supplementary material for: Multiomic analysis of monocyte-derived alveolar macrophages in idiopathic pulmonary fibrosis
Source: J Transl Med. 2024 Jun 27;22:598. doi: 10.1186/s12967-024-05398-y (PMC11209973; doi:10.1186/s12967-024-05398-y)
Supplement: Supplementary file 2 — Supplementary Material 2 [file 12967_2024_5398_MOESM2_ESM.pdf]

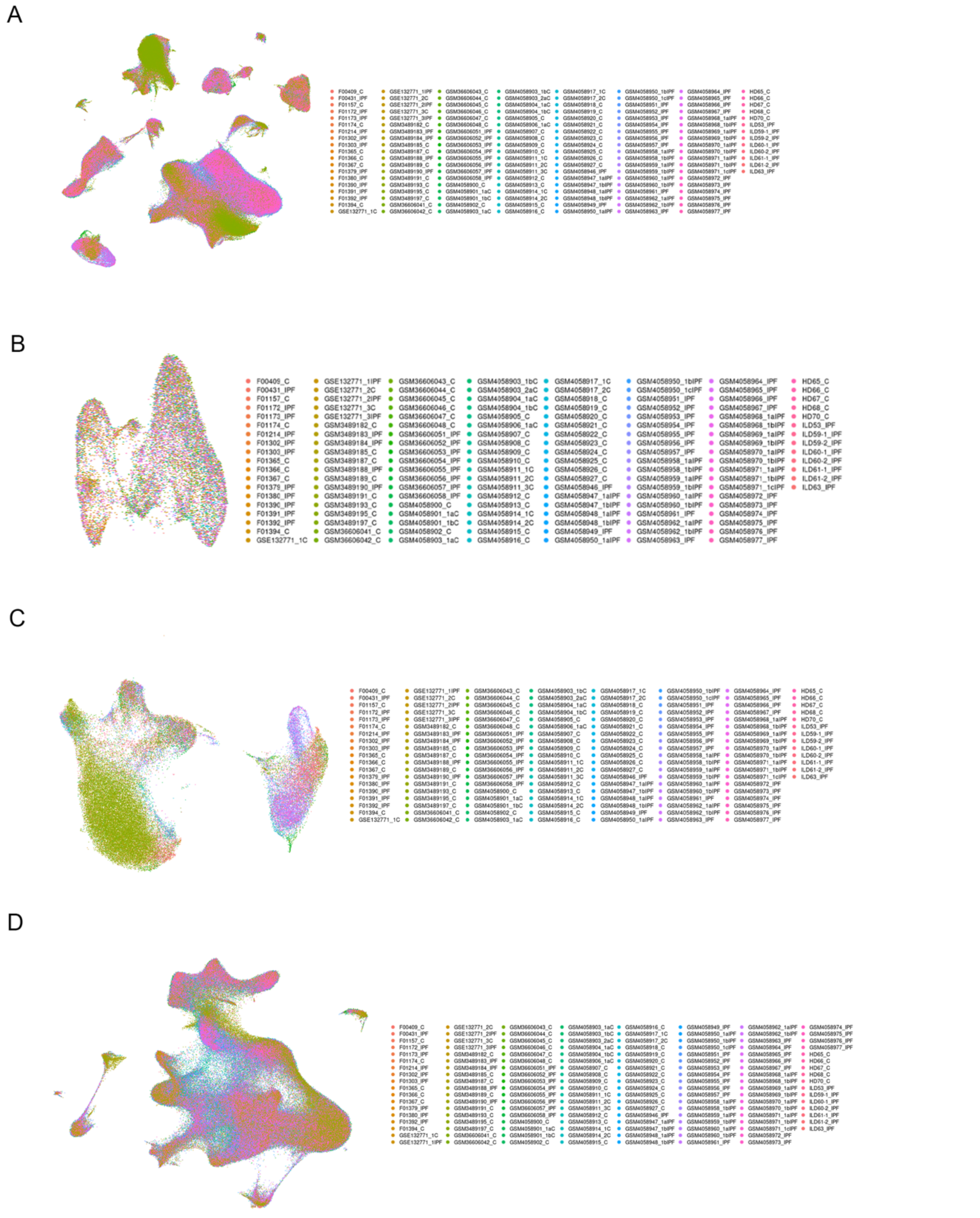

Fig. S1

UMAPs of Four Main Cell Types (A), Stromal Cells (B), Epithelial Cells (C), and Immune Cells (D) Coloured by Sample

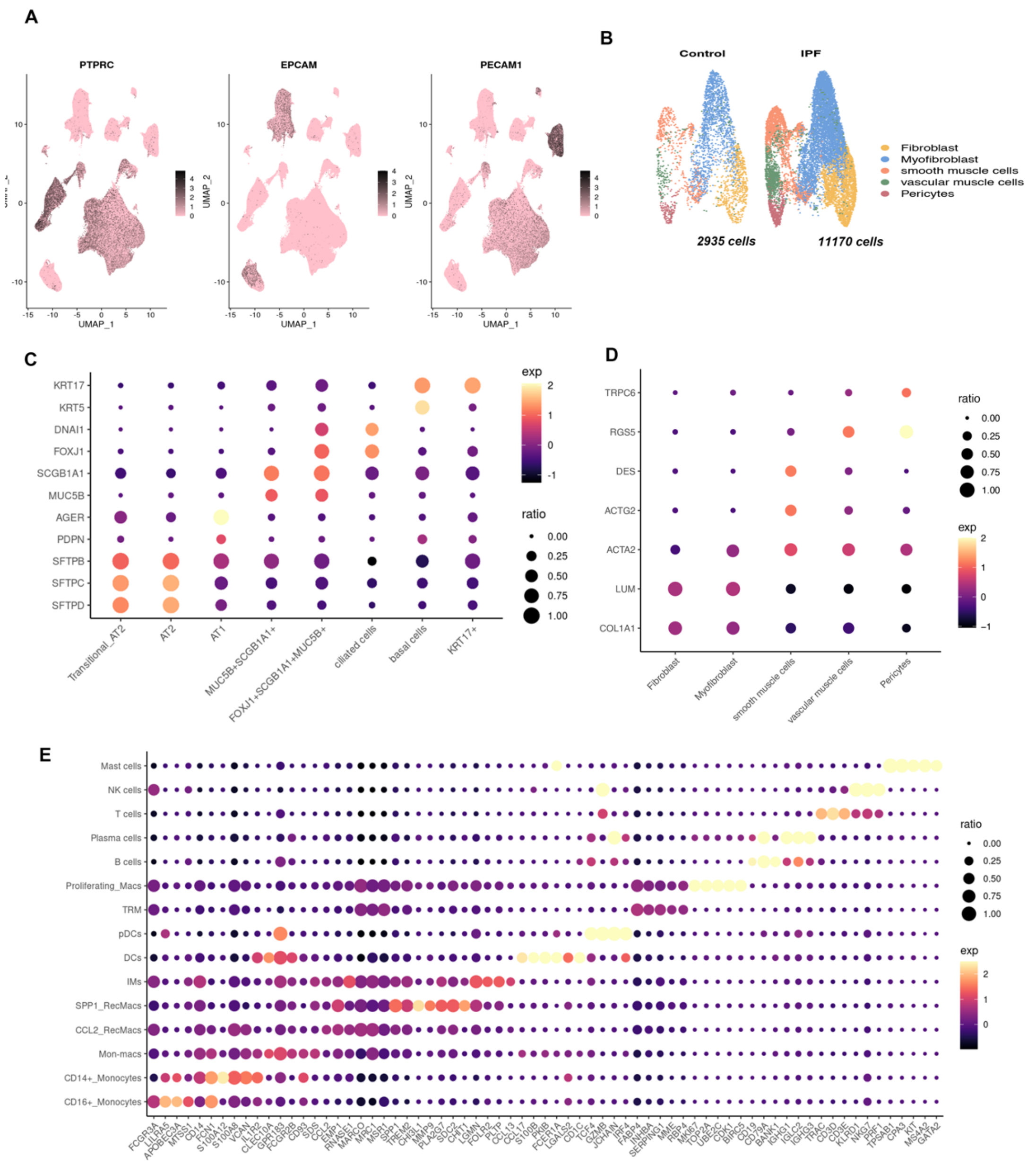

Fig. S2

Maker *G*ene *E*xpression for *C*ell *C*lusters in the Idiopathic Pulmonary Fibrosis Lung Tissue *A*tlas

Featureplots of PTPRC, EPCAM, and PECAM1 (A). Epithelial cell type (C), stromal cell type (D), and immune cell type (E) makers. UMAP clusters of stromal cells split by the control group and the idiopathic pulmonary fibrosis group.

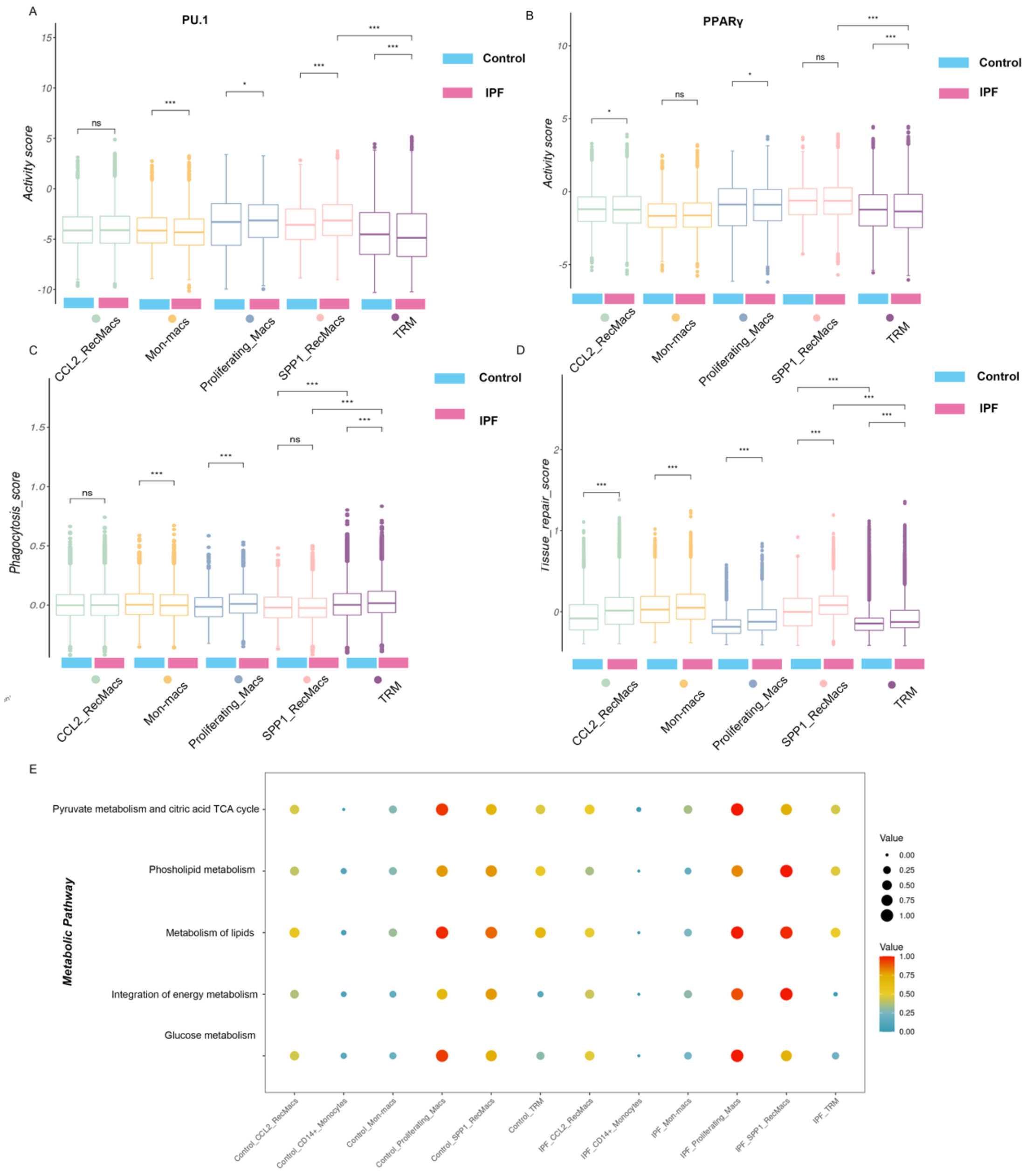

Fig. S3

Differences of Mo_AMs and TRMs in Transcription Factor Activities, Phagocytosis, Tissue Repair, and Metabolic Profiles

Transcription factor activity of PU.1 (A) and PPARγ (B) in Mo_AMs and TRMs using the DorothEA database. Inferring phagocytosis (C) and tissue repair (D) in Mo_AMs and TRMs using the Addmodulescore function. The lipid and energy metabolism analysis in Mo_AMs using the scMetabolism package.

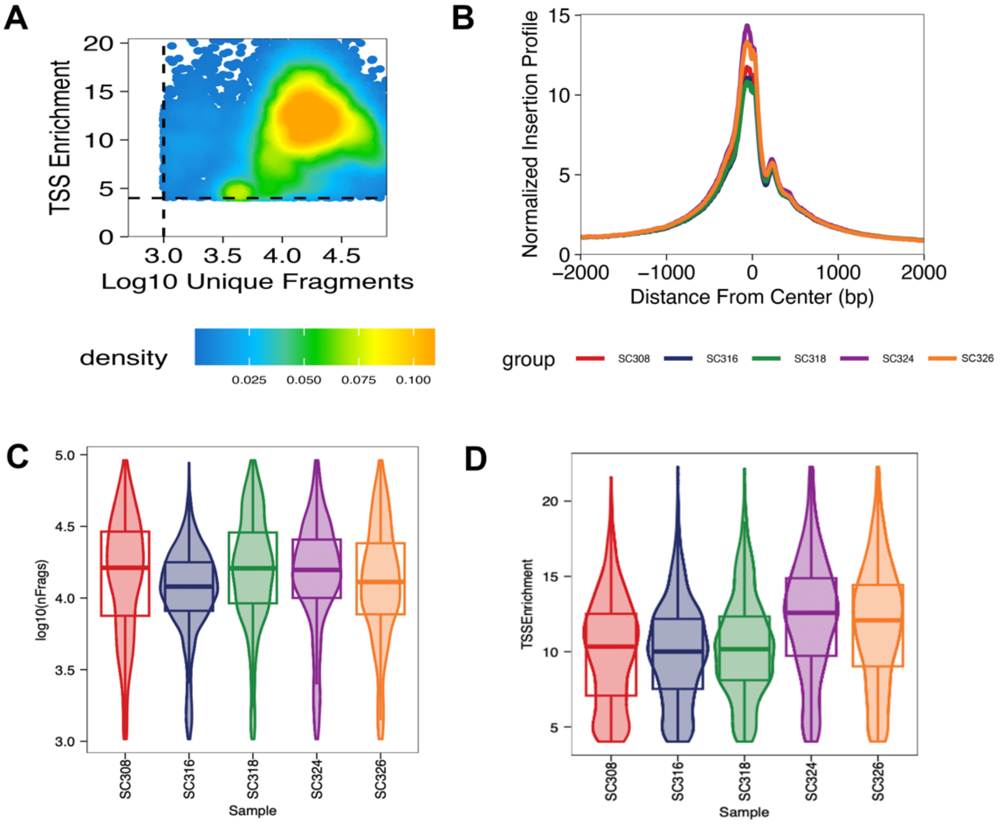

Fig. S4

Quality Control of the scATACseq

Filtering plots showing transcription start site enrichment and number of unique fragments (A), distribution plot of the distance from centre for each fragment (B), violin plots of fragments (C), and transcription start site enrichment (D) split by sample.
